# Genetic diversity of *Schistosoma haematobium* parasite IS NOT associated with severity of disease in an endemic area in Sudan

**DOI:** 10.1186/1471-2334-14-469

**Published:** 2014-08-27

**Authors:** Nagla Gasmelseed, Nhashal E Karamino, Mohammed O Abdelwahed, Anas O Hamdoun, Ahmed E Elmadani

**Affiliations:** Department of Molecular Biology, National Cancer Institute, University of Gezira, Wadmedani, Sudan; Department of Medical Imaging, National Cancer Institute, University of Gezira, Wadmedani, Sudan

**Keywords:** *S. haematobium*, Genetic diversity, Ultrasound, Severity, Gezira State, Sudan

## Abstract

**Background:**

Over 650 million people globally are at risk of schistosomiasis infection, while more than 200 million people are infected of which the higher disease rates occur in children. Eighty three students between 6-20 years (mean 12.45 ± 3.2) from Quran School for boys in Radwan village, Gezira state were recruited to investigate for the relationship between the genetic diversity of *Schistosoma haematobium* strains and the severity of the disease.

**Method:**

*Schistosoma haematobium* infection was detected by filtration of urine. Ultrasonography was done on each study subject, while PCR technique was used for genotyping via random amplified polymorphic DNA (RAPD) with A01, A02, A12, Y20 and A13 primers. A01 primer gave three different genotypes (A01-1, A01-2 and A01-3).

**Results:**

About 54.2% (45/83) were *S. haematobium* egg positive by urine filtration. On assessment of the upper and lower urinary tract by ultrasound technique, 61.4% (51/83) were positiveand73.3% (60/83) samples were PCR positive. No significant difference was found when comparing the three different genotypes with severity of the disease.

**Conclusion:**

This study concludes that no association was found between the different genotypes of *S.haemtobium* and the severity of the disease. Examination of more samples from different areas to identify any possible differences between the parasites genes and disease severity was recommended.

**Electronic supplementary material:**

The online version of this article (doi:10.1186/1471-2334-14-469) contains supplementary material, which is available to authorized users.

## Background

The genome of *Schistosoma* is approximately 270 Mbp [1], which is considerably about one tenth the size of the human genome. It is estimated that the *S. mansoni* genome has a GC content of 34% [2], with 4-8% highly repetitive sequence, 32-36% middle repetitive sequence and 60% single copy sequence [1]. Numerous highly or moderately repetitive elements have been identified and their occurrence within existing sequence datasets also indicates that the genome contains at least 30% repetitive sequence [3]. With genetic crossing occurring between adult worms, heterogeneity of an infection may strongly influence development of new variants of the parasite [4]. Recent studies using randomly amplified polymorphic DNA (RAPDs) markers have found multiple genotype infections by *S. haematobium* in the *Planorbidae* intermediate host [5, 6]. RAPDs mainly segregate as dominant markers with heterozygous and homozygous dominant individuals having the same banding pattern at a locus [6], while homozygous recessive individuals have no band at that locus. Statistical measures taking this limitation into account have been developed in order to derive population parameters describing heterozygosity, gene flow, linkage disequilibrium, and other factors [7]. Randomly amplified polymorphic DNA (RAPD) studies of *Schistosoma mansoni* infection in naturally infected rodent species revealed even more genetic diversity per infection with as many as 28 specific genotypes per rat [8].

Although few specific primers for polymorphic regions exist, RAPD-polymerase chain reaction (PCR) technology has made it possible to conduct population-based studies of schistosomes with little prior sequence information [9]. RAPD primers have been used in several studies to examine the genetic diversity among populations of schistosomes in snails [10, 11] and have proved valuable despite the assumptions necessary for their interpretation [12]. They have demonstrated that, with *S. haematobium*, it is feasible to sample a population of parasites conveniently and arrive at estimates of the frequency of various alleles using RAPD–PCR. The method of schistosome sampling, through single genetic drift, and through limited numbers of infected snails, snail mortality, does play a role, increasing the numbers of snails for haematobium [13]. Knowing the extent of parasite gene flow and the nature of any barriers to such flow will be important in predicting the likely washing spread of drug resistance genes, if they appear [13]. RAPD primers survey a large number of loci throughout the entire genome and have proven useful in characterizing both inter- and intraspecific relationships [14, 15]. As very little DNA is required for such analyses, cercarial or miracidial stages of schistosomes can be used as a source of genetic material, thus minimizing selection caused by passaging through unnatural hosts [13, 16].

Distribution of *S.haematobium* in Sudan in several irrigation schemes have been constructed e.g. Gezira, Rahad Agriculture Scheme, Ginaid, AL Gerba, Kenana and other sugar cane schemes [17], also found in small part of Blue Nile, southern of Sudan [18]. Little is known about the extent of genetic diversity of *S. haematobium* within its definitive host, humans. Understanding the genetic structuring of populations at each stage of the life cycle is essential to account for the creation of diversity and its maintenance in natural populations of parasites [19]. Genetic variability among parasite populations is an important factor in their potential for producing harmful effects on the human populations. Since damage from schistosome infections is so closely linked to the immune reaction to parasite eggs deposited in tissue, diversity of this infection may play an important role in development of pathology with heterogeneous versus homogeneous infections resulting in different clinical outcomes. Genetic differences may also lead to some strains being innately more immunogenic or fecund than others [20]. The aim of this study was to identify if there is any relationship between the genetic diversity of *S.haematobium* and pathology of the disease in school children in Gezira State, Sudan.

## Methods

### Study area

This study was conducted in Roudwan village, located in the south west of Wadmedeni city. This village is a part of Gezira irrigation scheme and surrounded by canals from the south and east. There is only one small health center and no school as most of the children study in Khalwa (Quranic schools). No safe water source is available, and the main source of water is from canals.

Quranic School (Khalwa) was selected for this study and the students came from different regions of Gezira state and other states of Sudan such as (Blue Nile state, White Nile state, Sennar, Gadarif). They stay up to 15 years to study the Quran. All the students were at risk of infection of schistosomiasis due to the daily exposure to the infection by swimming and daily activities in canal water. Eighty three students aged 6-17 years old were recruited from Hilt Radowan (Khalwa).

### Urine filtration technique

Urine sample were collected from each students and 10 ml examined by filtration method [21]. *S.haematobium* eggs were detected and egg count recorded as number of egg/10 ml. The remaining urine was centrifuged and the deposit kept at -80°C as preparation for DNA extraction. The students were weighted and all positives for *S. haematobium* infections were treated with Praziquantel.

### Ultrasound examination

Ultrasonographic examination was done by expert radiologist on each student. Transabdominal ultrasonographic examination of the urinary tract was performed by Aloka (5100) with a 3.5 MHz convex probe and Shimadzu with convex probe 2-5 and 5-10 MHz on all the study subjects after adequate filling of the bladder. Urinary tract and kidneys were examined according to WHO standards [22]. The bladder was evaluated using a 3.5 MHz sector scan in three directions. Bladder wall thickness was measured, and mucosal irregularities, masses or pseudopolyps were classified as proposed by the WHO workshop [22]. Irregularities of the bladder wall or thickening were considered pathological in the case of more than 0.5 cm in thickness. Ureter dilation was evaluated in the retrovesical region and if indicated this examination was repeated after micturition to exclude vesico-ureteral reflux. The severity of *S.haematobium* divided according to the size of bladder wall thickening irregularity, calcification, nodularity, dilatation and ureter thickness, nodularity, dilatation, calcification and kidney dilatation, and echogencity. Classification of severity was done by ultrasonographicas normal or abnormal finding in upper and lower urinary tract.

### Genotyping of *S. haematobium*DNA using random amplified PCR reaction (RAPD)

DNA extraction was done for all collected samples using QIAamp DNA Mini kit (Germany). Genotyping of *S. haematobium* DNA using Random Amplified PCR Reaction (RAPD) test the amplification conditions were based on the original method described by Williams et al (1990). In this study five primers were used for genotyping of *S. haematobium* strain (A01, 5′-CAGGCCCTTC-3′;A02, 5′-TGCCGAGCTG-3′;A12, 5′-TCGGCGATAG-3′;A13, 5′-CAGCACCCAC-3′;Y20,5′-AGCCGTGGAA-3′;), primers were selected according to Shiff et al (2000). Amplification reaction was carried out in a final volume of 25 μl and the PCR product determined on 4% polyacrylamide by electrophoresis.

### Ethical approval

Ethical clearance was obtained from National Cancer Institute Research Ethical committee NCI-REC University of Gezira. Prior to conducting the study, aims and all information about the study was explained to community during meetings. Written consent was obtained from the local leaders. All infected subjects were treated immediately after diagnosis.

### Statistical analysis

Data was analyzed using SPSS version 16 statistical package. Descriptive, correlation and chi square analysis done in this study.

## Results

**Figure 1:**
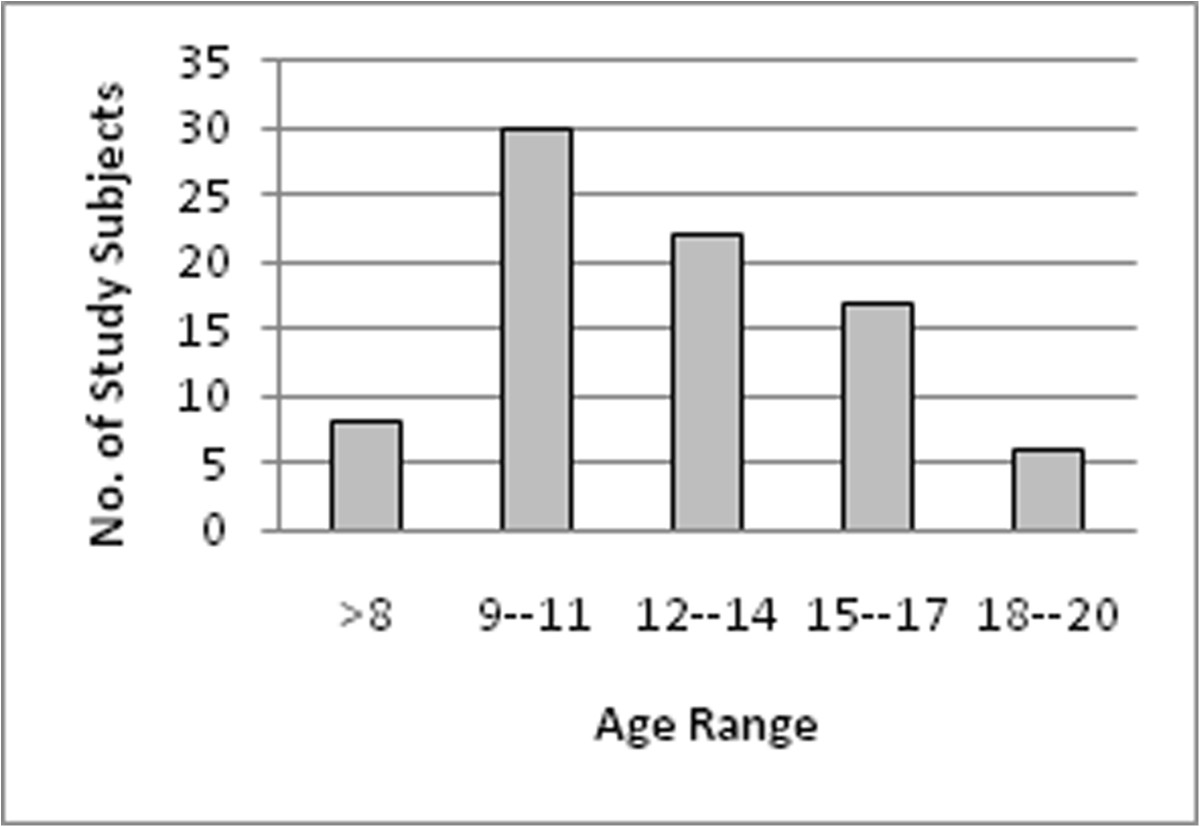
**Distribution of the study subjects by age range, N = 83.**

**Figure 2:**
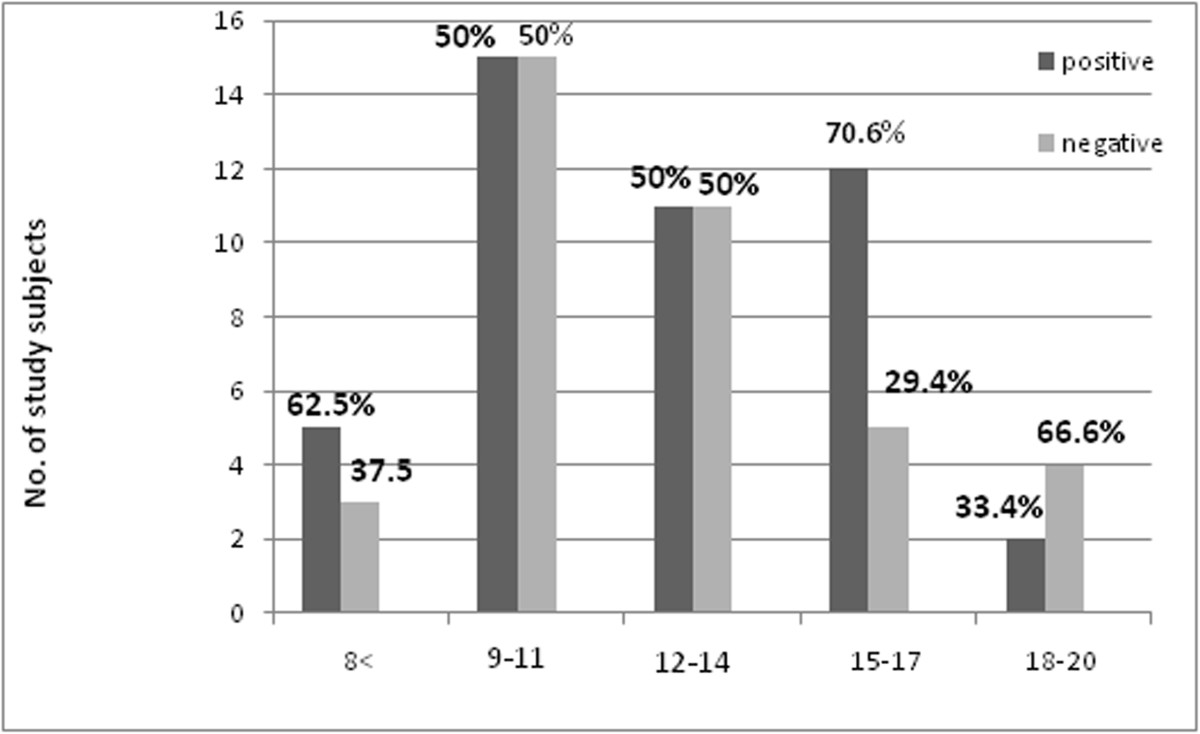
**Shows the results of study subjects age groups by filtration technique, N = 45.**

All of the study subjects were males; the mean age was 12.45 ± 3.2 with age range between 6 - 20 years. The majority of age groups were between 9-11years, 36.1% (30) followed by 12-14years 26.5% (22) as shown in Figure 1. About 54.2%(45) of the students were positive to *S.haematobium* by filtration. The stratification of the age range showed that the high positive frequency was 18% (15) in the age range (9-11 years), as in Figure 2.

**Figure 3:**
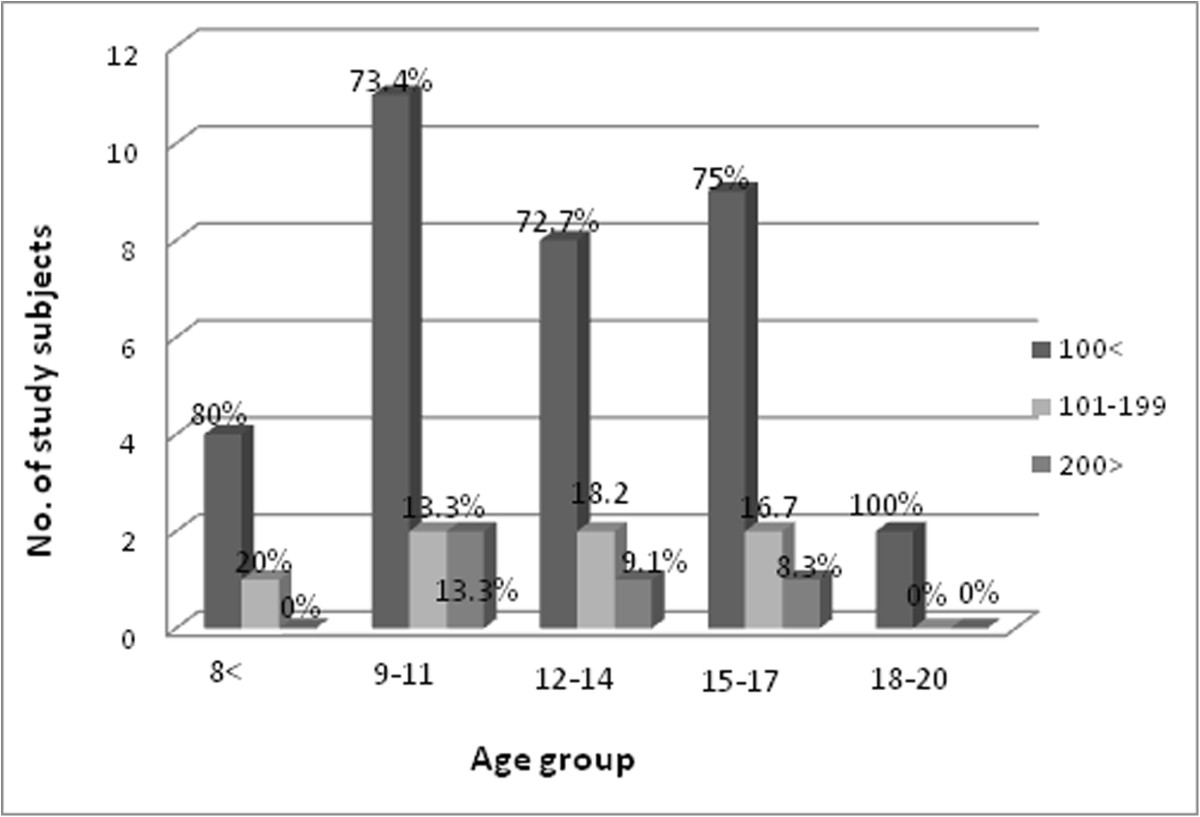
**Shows result of** ***S.haematobium*** **egg count by filtration technique, of 45 study subjects.**

The intensity of infection was classified as egg count <100 eggs recorded as low level infection and between 101-199 eggs as moderate and >200 eggs as high intensity of infection [23]. Forty three out of 45 (75.5%) had a low intensity of infection while only 9.0% (4) had a high intensity (Figure 3).

### Detection of *S.haematobium*by Ultrasound

**Figure 4:**
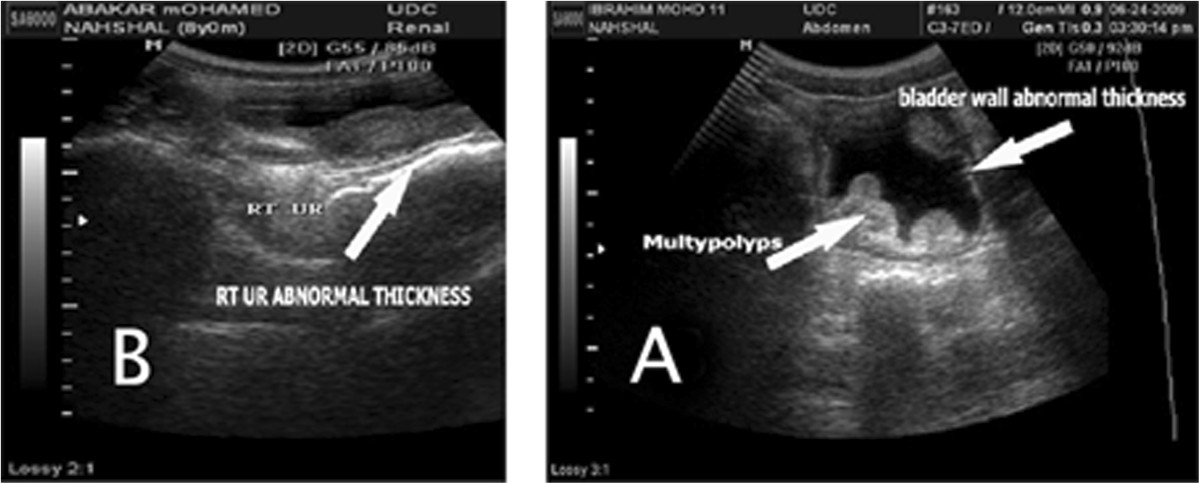
**Shows Ultrasound Image. A**: shows thickness on wall of bladder, polyps and calcification **B**: shows thickness of ureter 6 mm and Ultrasonographic examination was done by experienced radiologist.

Eighty three of the Khalwas’ students were diagnosed by ultrasound. The diagnosis included upper urinary tract (Kidneys and ureters) and lower urinary tract (urinary bladder). About 85% (51) of study subjects had pathological changes. Those with abnormalities were 85.9% (49); abnormal wall thickness 56.8% (29) multiple nodularities and 3.9% (2) one polyp as shown in Figure 4A, and1.9% (1) wall bladder calcifications,11.7% (22) abnormal ureteric thickness (Figure 4B) and 3.9%(2) echogenic particles (Table 1).

**Table 1:** **Ultrasound finding of the study subjects by age range**

Age range	Total (%)	Thickness (%)	Bladder mass		Bladder wall	Ureter thickness (%)	Echogncity (%)
			One polyp (%)	Multiple (%)	Calcification (%)		
>8	5 (9.8)	5 (10.2)	1 (50)	3 (10.3)	0	0	0
9-11	21 (41.2)	20 (40.8)	0	14 (48.3)	0	7 (31.8)	0
12-14	16 (31.4)	15 (30.6)	1 (50)	9 (31.0)	1 (100)	8 (36.4)	0
15-17	7 (13.7)	7 (14.3)	0	2 (6,9)	0	5 (22.7)	1 (50)
18-20	2 (3.9)	2 (4.1)	0	1 (3.4)	0	2 (9.1)	1 (50)
Total (%)	51 (100%)	49 (100)	2 (100)	29 (100)	1 (100)	22 (100)	2 (100)

### Molecular genotyping of *S.haematobium*strain

**Figure 5:**
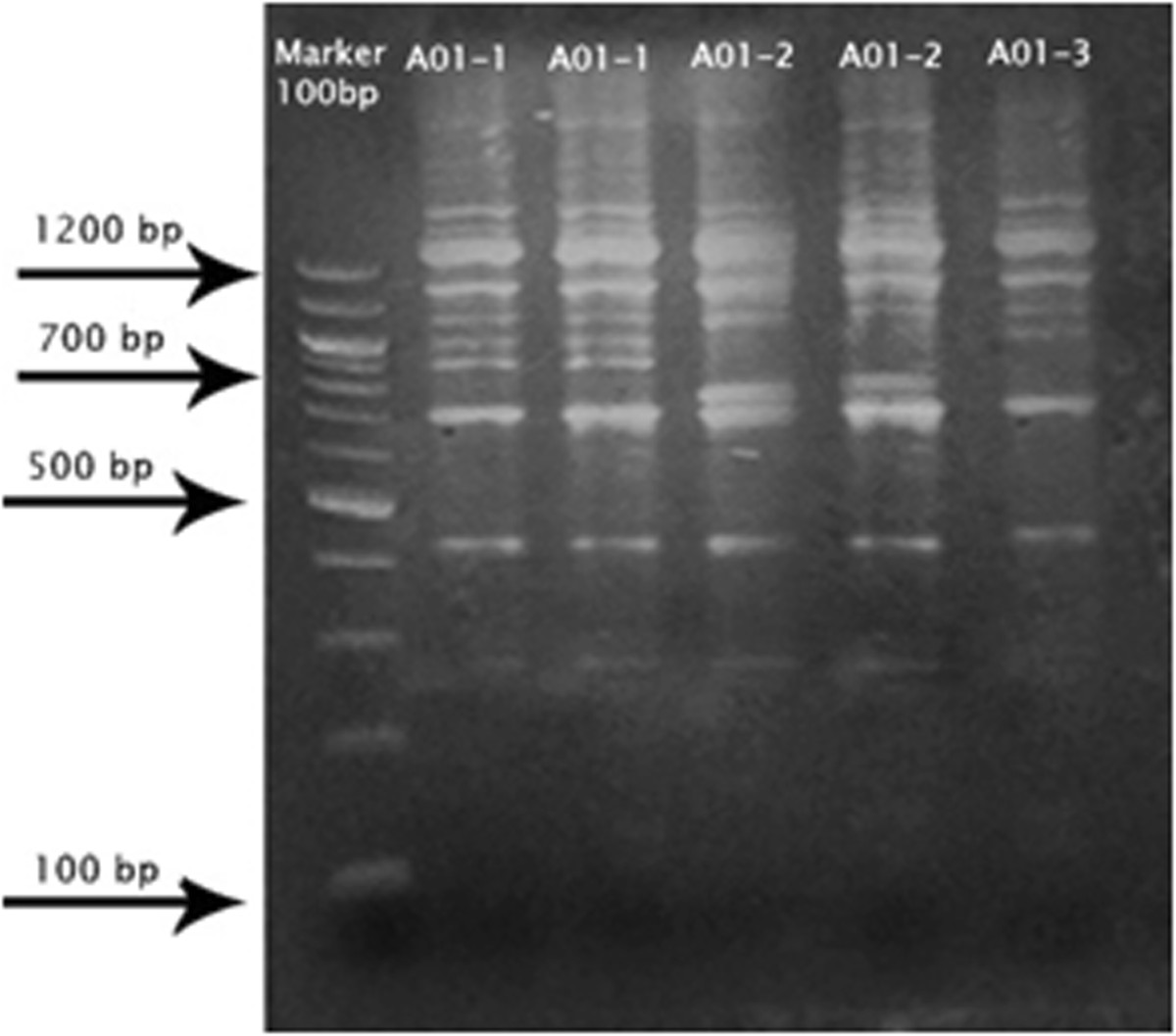
**Shows** ***S.haematobium*** **genotypes for A01 primer in polyacrylamide gel.**

PCR was done for all urine samples using RAPD test 72.3% (60) study subjects had a PCR product for A01 primer, while no product was detected for the other primers (A02, A12, A13, Y20). A01 primer showed three different genotypes mentioned as genotype (1, 2, and 3) in polyacrylamide gel using 100 bp DNA marker polymorphic band in marker 700, 800, 900, 1100 bp (Figure 5).

### *S.haematobium*infection severity and the genotypes

72.3% (60) were positive by PCR; 80% (48) were genotyped A01- genotype 1, 15% (9) were genotype A01-genotype2 and 5% (3) were genotype A03-3. Correlation between severity and genotype of *S.haematobium*, genotype A01 (genotype1) recorded;33.3% (16) study subjects were mild, 20.8% (10) had moderate severity and 20.8% (10) had severe feature of the disease. Genotype A01-2 was recorded 44.4% (4) moderate and 22.3% (2) were severe. There is no significant correlation between Severity of *S.haematobium* and the genotypes Pearson Chi-Square = 24, *P- Value* = 0.004 (Table 2).

**Table 2:** **Shows association between severities and genotypes of** ***S.haematobium***, **N = 60**

	Severity of the disease				Total%
Genotype A 01	Normal (%)	Mild (%)	Moderate (%)	Severe (%)	
Genotype 1	11 (73.3)	17 (94.4)	10 (71.4)	10 (76.9)	48 (80)
Genotype 2	3 (18.7)	0 (00)	4 (28.6)	2 (15.4)	9 (15)
Genotype 3	1 (6.7)	1 (5.6)	0 (00)	1 (7.7)	3 (5)
Total	15 (100)	18 (100)	14 (100)	13 (100)	60 (100)

Forty five study subjects were positive by PCR and filtration; 87% (39) were genotype A01- 1, 9% (4) were genotype A02-2 and 5% (2) were genotype A03-3. Correlation between infection severity and genotype of *S.haematobium*, genotype A01 (type1) recorded; 25.6% (10) normal and 38.4% (15) mild, 18% (7) had moderate infection severity and 18% (7) had severe clinical feature. Genotype A01-2 had 50% (2) normal and 25% (1) were severe, while 25% (1) were moderate. There is no significant correlation between Severity of *S.haematobium* and the genotypes Pearson Chi-Square = 4, p = 0.6, (Table 3).

**Table 3:** **Shows association between severity and positive study subject by filtration method and positive by PCR, N = 45**

	Severity of the disease				Total (%)
Genotype A 01	Normal (%)	Mild (%)	Moderate	Severe	
Genotype 1	10 (83.3)	15 (93.8)	7 (87.5)	7 (77.8)	39 (87)
Genotype 2	2 (16.7)	0	1 (12.5)	1 (11.1)	4 (9)
Genotype 3	0 (00)	1 (6.2)	0	1 (11.1)	2 (5)
Total	12 (100)	16 (100)	8 (100)	9 (100)	45 (100)

Fifteen study subjects were positive by PCR and negative by filtration; 9/15 was genotype A01- 1, while 5were genotype A02-2 and 1were genotype A03-3. Correlation between infection severity and genotype of *S. haematobium*, genotype A01 (type1) recorded;11.1% (1) normal and 22.3% (2) mild, 33.3% (3) had moderate infection severity and 33.3% (3) had severe clinical feature. Genotype A01-2 had 60% (3) moderate and 20% (1) had severe infection, while 20% (1) were normal. There is no significant correlation between infection severity of *S.haematobium* and the genotypes Pearson Chi-Square = 6, p = 0.3 (Table 4).

**Table 4:** **Shows association between severity and negative study subject by filtration method and positive by PCR, N = 15**

	Severity of the disease				Total (%)
Genotype A 01	Normal (%)	Mild (%)	Moderate (%)	Severe (%)	
Genotype 1	1 (33.3)	2 (100)	3 (50)	3 (75)	9 (60)
Genotype 2	1 (33.3)	0 (00)	3 (50)	1 (25)	5 (33)
Genotype 3	1 (33.3)	0 (00)	0 (00)	0 (00)	1 (6.4)
Total	3 (100)	2 (100)	6 (100)	4 (100)	15 (100)

## Discussion

Schistosomiasis affects approximately 200 million people, mainly in rural areas of developing countries, with an estimated 79 million people at risk of the disease [24]. Both *S. haematobium*and *S. mansoni* are present in Sudan. Schistosomiasis is found in many different areas in Sudan especially in Gezira, Rahad, Kenana and other irrigation schemes [25, 26]. The dynamics of the transmission are necessarily complicated and subject to considerable variation due to many factors influencing the common environment, the behavioural patterns of the definitive host and the bionomic of the intermediate host.

This study was carried out in Radwan village in Gezira state, Sudan. The village is surrounded by canals without sufficient safe water supply. *S. haematobium* started increasing in the last decade after control of *S.mansoni* in Gezira area [17]. Schistosomiasis affects males more than females (unpublished report 2011), since males are more exposed to the water supply than females (culturally).

In this study 51% of study subjects were found to have abnormal pathological conditions including; 85.9% (49) abnormal wall thickness 56.8%(29) multiple nodularity and 3.9% (2) one polyps and 1.9% (1) wall bladder calcifications. When compared to a similar study from Mali [27] more irregular bladder wall were the most frequently diagnosed abnormality in 3.4% of children. Study from Nigeria [28] reported 71% abnormal pathological conditions; abnormal wall thickness (55.8%) while in our study 56.8%, irregular bladder wall (27.9%), masses (23.3%), pseudo polyps 2 (4.7%) compare with 5.8% in this study. These studies reported similar findings that most abnormal urinary bladder wall recorded in children below 15 years old. In this study there was no correlation between ultrasound abnormal urinary tract findings and intensity of infection (P = 0.21). This is contrary to the study from Nigeria [28] that reported abnormal pathology slightly more common in the study subjects with heavy infection than those with light infection.

Another study conducted in southern Nigeria identified about 6.7% and 1.7% of the patients had the right pelvis and left pelvis of their kidney moderately dilated, respectively [29]; in contrast to this study where no kidney abnormalities were found probably due to duration of infection in the study participants.

Schistosomiasis diagnosis by PCR results recorded 72.3% (60) while schistosomiasis diagnosis by filtration methodsrevealed 54.2% (45), this may be due to presence of a small number of eggs that are difficult to diagnose by the microscope. Many studies confirmed the sensitivity of PCR to detect egg of *S.haematobium* in urine to be more sensitive than filtration technique. A study performed in Ghana using PCR methods for diagnosis reported sensitivity 100% and specificity at 89% [30], in contrast using PCR in this study, sensitivity was 100% and specificity was 60.5%. It is well known that sensitivity of PCR to diagnosis *S.haematobium* is more sensitive than filtration; this was indicated by 15 study subject in this study found negative by filtration while they were positive by PCR. But the study from northern Senegal showed significant correlation with microscopic egg counts both for *S. mansoni* in stool and *S. haematobium* in urine. They found that *Schistosoma* detection rate of PCR (84.1%) was similar to that of microscopy performed on duplicate stool samples (79.5%) [31], that may be due to high intensity of infection that made the sensitivity of filtration similar to PCR.

In this study PCR was done by Using RAPD test 72.3% (60) study subjects had a PCR product for A01 primer, A01 primer showed different three genotypes (1, 2, and 3) 80% (48) were genotype A01-1 while 9/60 (15%) genotype A01 - 2 and 5% (3) genotype A01-3, in polyacrylamide gel. Study done by [13] RAPD fragments of an inbred Egyptian strain of *S. haematobium* generated by primers G17 and A02, using miracidial isolate from a particular host. Differences in alleles were recorded among the 37 variable bands. In this study, only three variable bands were recorded, and polymorphic region was between 700 and 1100 bp. The small number of variation alleles in this study refer to the samples collection from children infected with schistosomiasis which transmitted to them from the same study area, while [13] study samples were collected from infected students coming from different regions infected with *Schistosoma*, this can increase the chances of finding more different polymorphic.

RAPD primers have been used in several studies to examine the genetic diversity among populations of schistosomes in snails [5]. Studies done by [5] at different sites in Bamako, on cercariae isolated from different snails and 47 different genotypes were recorded from 414 schistosome individuals. Ten primers (A01, A02, A10, A13, A19, G09, G11, G17, G19, G18) were used but only three primers were detected (A01, A10, G19), In this study 5 primers (A01, A02, A12, Y20 and A13) were used but only one primer was detected A01,the results were consistent with the study conducted by [5] with respect to genotype A01, where it was detected in the two studies, while genotype A02 and A13were used but did not give any results. Primer A01 revealed two polymorphic bands in marker 1100 bp, while in our study other variability in marker between 700 and 900 bp was noted. That means the findings show more different genotype when more than five primers are used or by increasing the study population that increases the chances of finding other genetic differences. In this study 46% of the subjects were positive by PCR with normal pathology by ultrasound. Most of these subjects belong to genotype A01, that may refer that the genotype is not aggressive enough to cause severe disease.

Morbidity manifestations have great role in diagnosis *S.haematobium* and genetic differences may lead to some strains being innately more immunogenic or fecund than others, these differences may play important role in severity features. There are few studies that found the relation between severity and genetic diversity [20]. In this study the association between the severity and genotypes was not found compared with a study from Zimbabwe that used ultrasonography to characterize the extent of urinary tract pathology of infected children, and random genetic markers to examine the relationship between genetic diversity of *S. haematobium* and clinical outcome. They found that parasite heterogeneity did not differ; allelic frequencies at eight loci differed significantly between the mild and severe groups. Parasite isolates were analyzed further using a modified cluster analysis that segregated the population into 13 clusters of associated genotypes. Three clusters were significantly over-represented in children with severe lesions. Although preliminary parasite genetic associations may be important in clinical outcome ([20]. In this study the genotype A01 type one was recorded as highest in severe 16.6% while 16.7% also were moderate, A01 type 2 was recorded 3.4% study sample severe and 6.7% moderate. There was no association between morbidity and different genotype as reported in a study from Zimbabwe. This may be due to *S. haematobium* infections differ under controlled circumstances, where factors such as exposure to parasites, infection intensity, type of parasite strains.

## Conclusion

Molecular characterization on *S.haematobium* proved that primer A01 with three different genotypes (polymorphic band between 700 and 1100 bp), is the most prevalent strain in study area. Difference in genotypes of *S.haematobium* may play a major role in severity features. However, molecular characterization of strain is needed to identify the type of strain in the area which may facilitate prevention and treatment. More samples from different parts of the state will be collected for future molecular study of the strains.

## Authors’ original submitted files for images

Below are the links to the authors’ original submitted files for images. Authors’ original file for figure 1 Authors’ original file for figure 2 Authors’ original file for figure 3 Authors’ original file for figure 4 Authors’ original file for figure 5
