# Supplementary figures and images for: Genetic diversity of Schistosoma haematobium parasite IS NOT associated with severity of disease in an endemic area in Sudan
Source: BMC Infect Dis. 2014 Aug 27;14:469. doi: 10.1186/1471-2334-14-469 (PMC4155082; doi:10.1186/1471-2334-14-469)

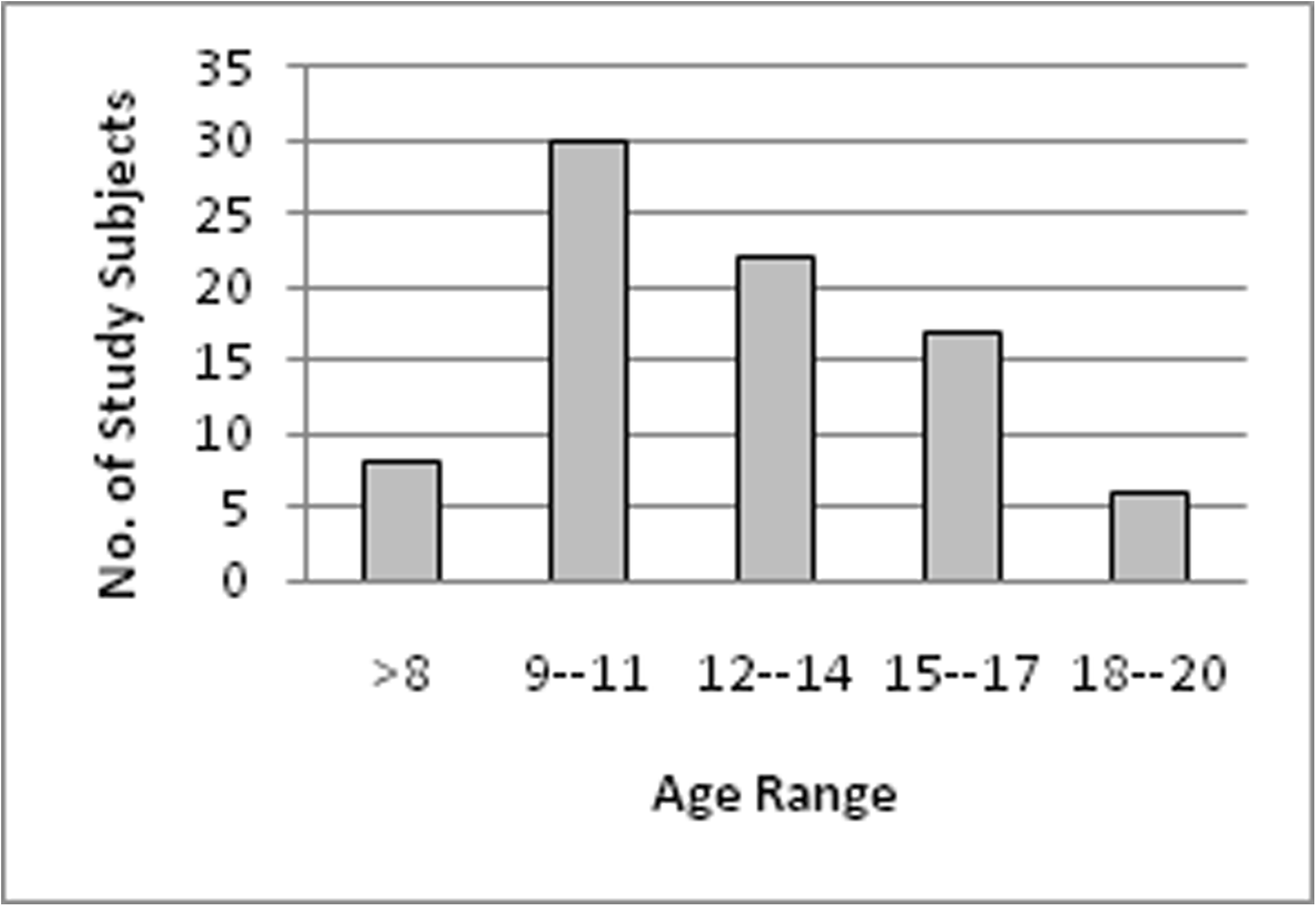

Supplement: Supplementary file 1 — Authors’ original file for figure 1 [file 12879_2014_3760_MOESM1_ESM.tif]

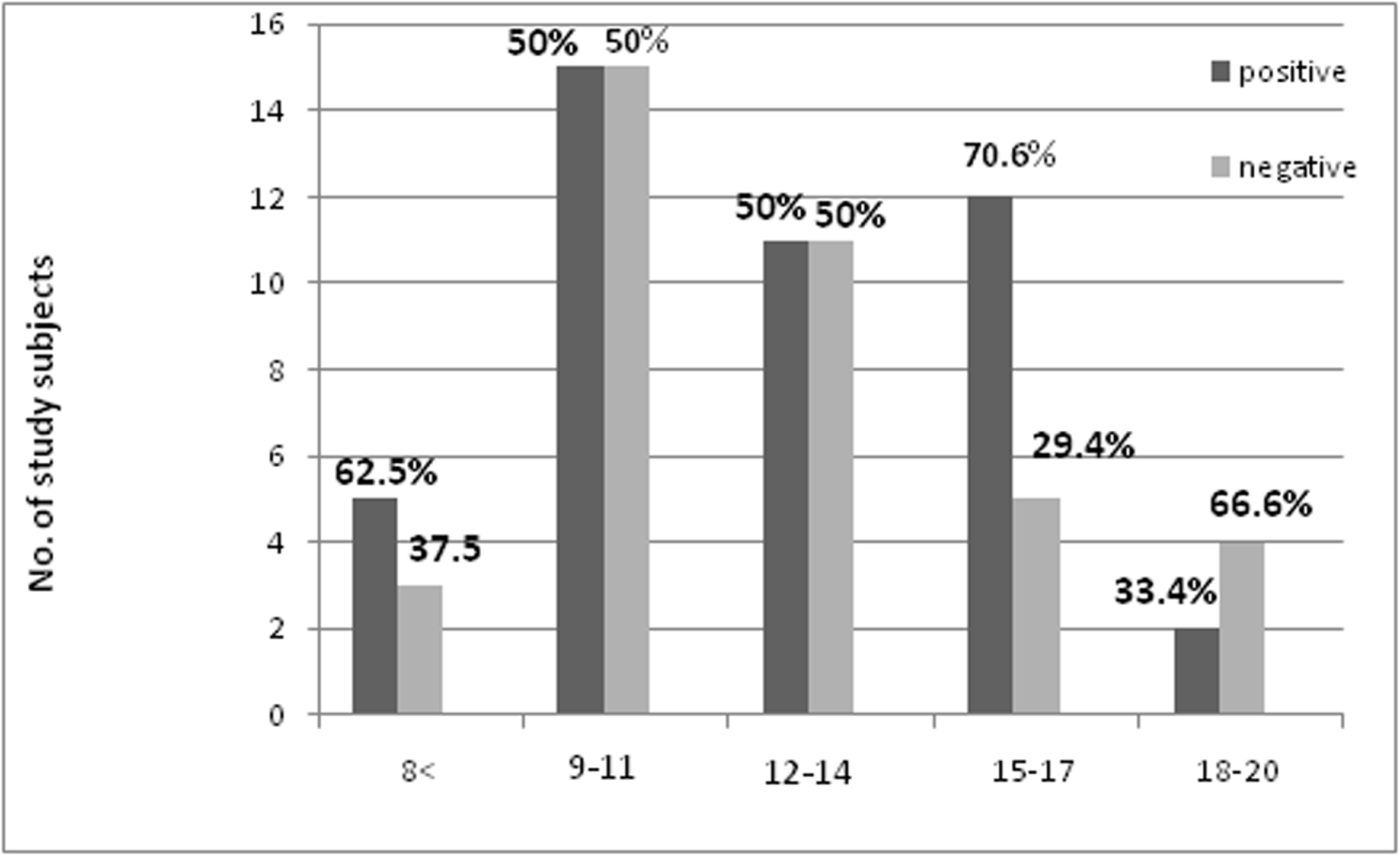

Supplement: Supplementary file 2 — Authors’ original file for figure 2 [file 12879_2014_3760_MOESM2_ESM.tif]

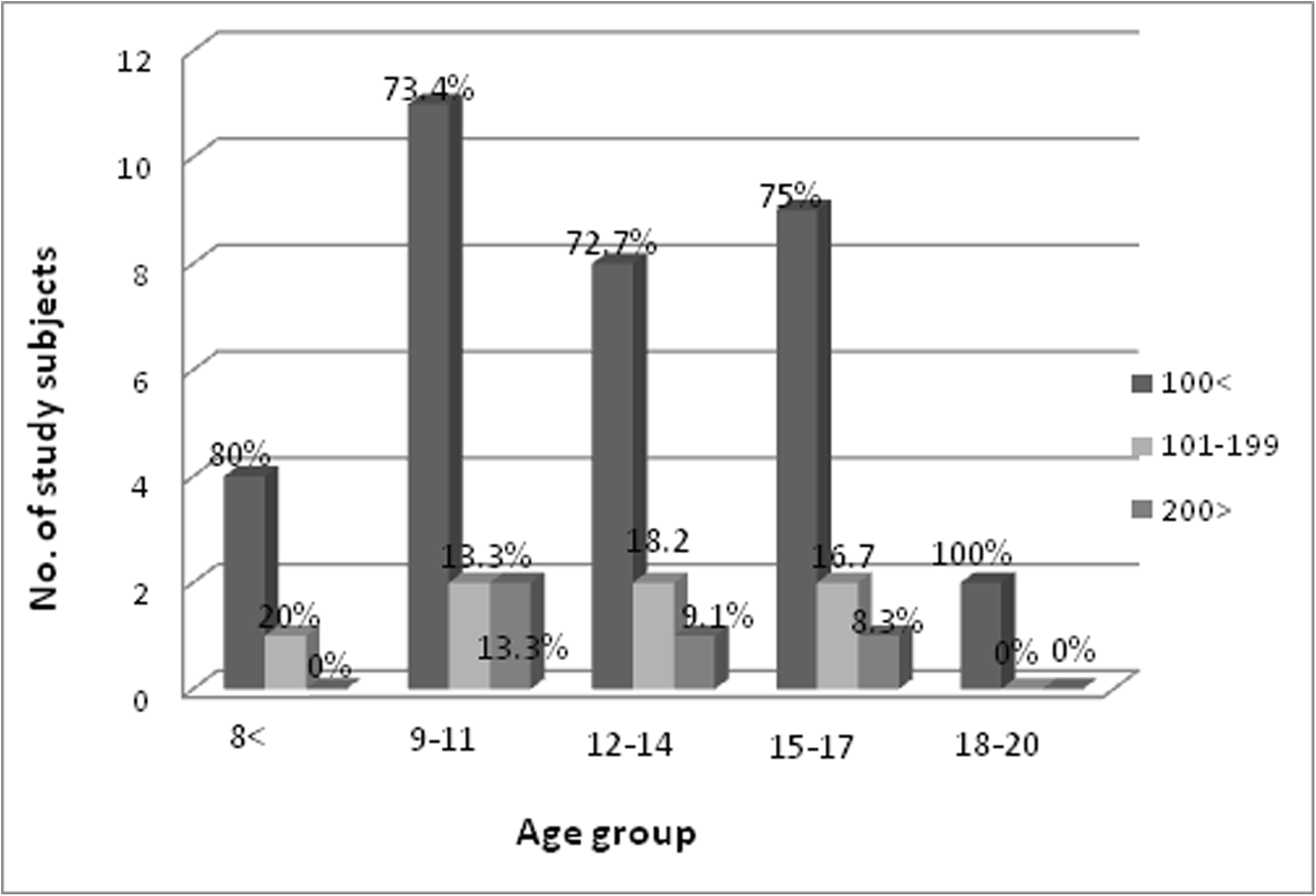

Supplement: Supplementary file 3 — Authors’ original file for figure 3 [file 12879_2014_3760_MOESM3_ESM.tif]

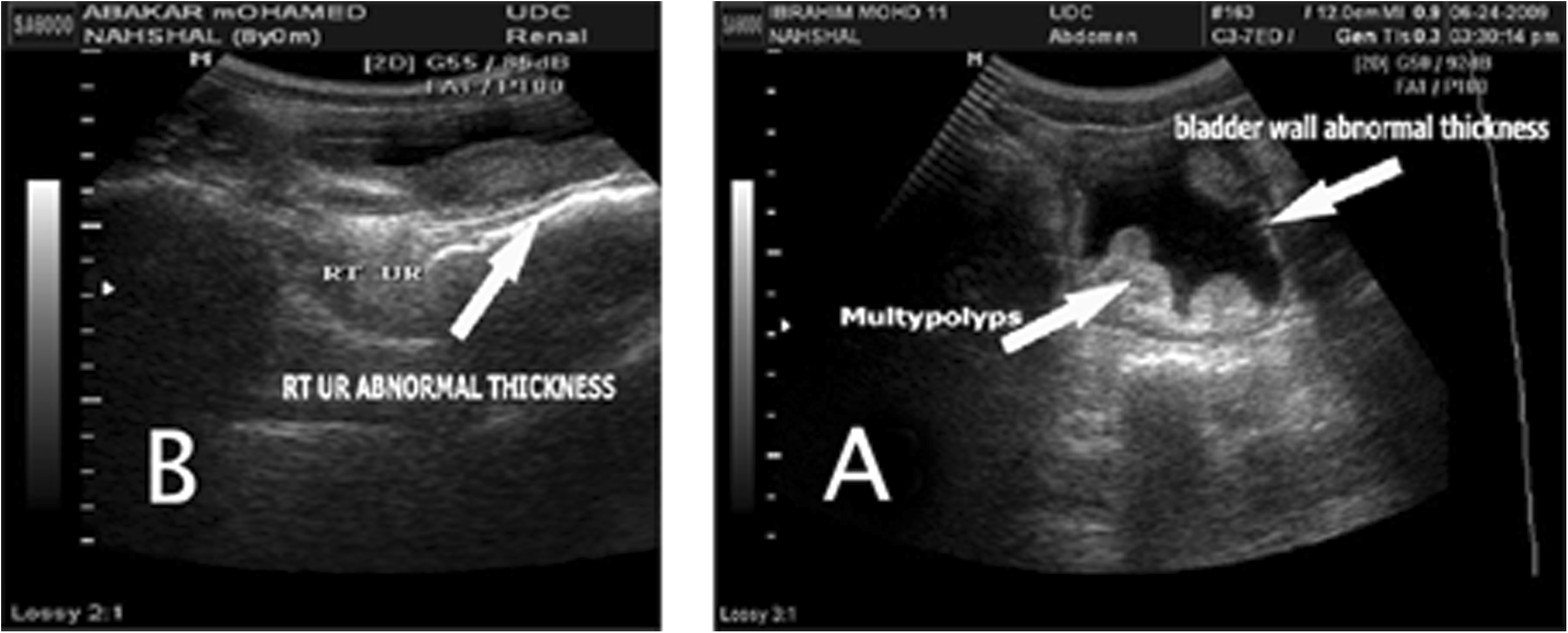

Supplement: Supplementary file 4 — Authors’ original file for figure 4 [file 12879_2014_3760_MOESM4_ESM.tif]

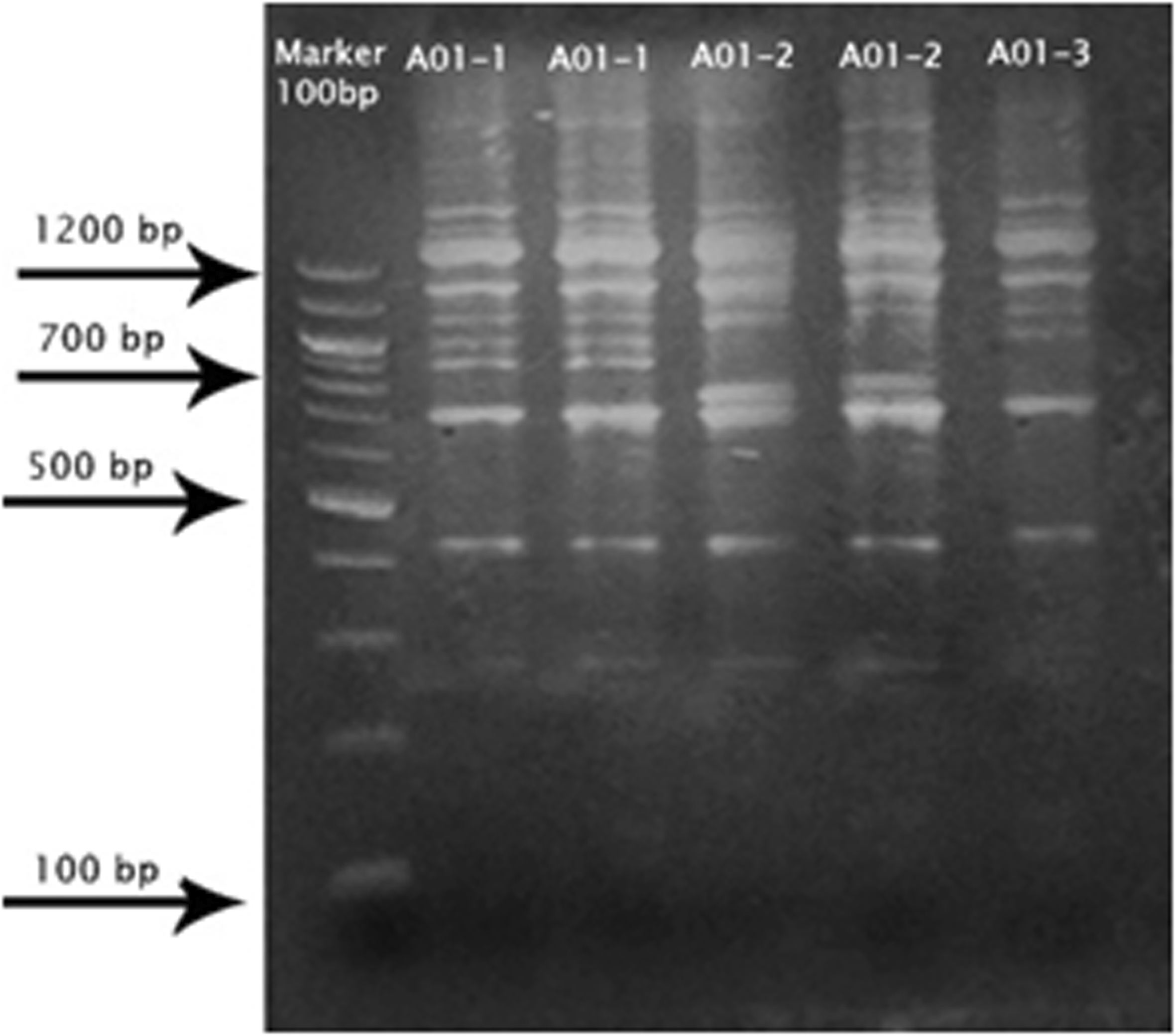

Supplement: Supplementary file 5 — Authors’ original file for figure 5 [file 12879_2014_3760_MOESM5_ESM.tif]
